# Development of simulation scenarios for surgeons’ non-technical skills evaluation

**DOI:** 10.1007/s44186-025-00390-6

**Published:** 2025-09-13

**Authors:** Nicholas E. Anton, Brittany Anderson-Montoya, Amy Holmstrom, Marian Obuseh, Yichuan Yan, Payton M. Miller, Wendy S. Li, Qais AbuHasan, Denny Yu, Dimitrios Stefanidis

**Affiliations:** 1 Department of Surgery, Indiana University School of Medicine, Indianapolis, IN 46202 USA; 2 Teladoc Health Inc., Harrison, NY USA; 3 Edwardson School of Industrial Engineering, Purdue University, West Lafayette, IN USA

**Keywords:** Simulation, Non-Technical Skills, Interpersonal Skills, Scenarios, Assessment, Behavioral Markers

## Abstract

**Purpose:**

The purpose of this study was to identify specific simulated scenarios and events for surgeons that discretely and effectively measure non-technical skills (NTS) constructs and appropriately measure NTS using specific behavioral anchors.

**Methods:**

Over the course of two rounds, experts in NTS accumulated a comprehensive list of simulated scenarios used at our institution to evaluate NTS. Utilizing a survey, experts were able to review scenarios, events, and behavioral anchors to evaluate their agreement with the appropriateness of the events and effectiveness of the anchors. Coefficient of variation (CV) was used to evaluate agreement between raters. The highest rated events were identified.

**Results:**

The CV between raters was moderate, and further inspection revealed that 86% of event ratings had discordance within ± 1 point. The highest rated events in terms of appropriateness and behavioral anchor effectiveness evaluated surgeons’ leadership and communication.

**Conclusions:**

In this study, experts identified three simulated events that isolate and discretely evaluate specific NTS among surgeons. In the future, these scenarios can be used to effectively evaluate surgeons’ NTS.

**Supplementary Information:**

The online version contains supplementary material available at 10.1007/s44186-025-00390-6.

## Introduction

Among healthcare workers, high-level non-technical skills (NTS) are imperative for the safe and effective provision of care to patients. NTS domains include interpersonal (e.g., communication and leadership) and cognitive skills (e.g., decision making and situation awareness) that are needed for all aspects of surgical performance [1]. Among surgeons, situation awareness reflects the maintenance of up-to-date understanding of the operating room environment throughout a procedure, decision-making refers to cognitive flexibility to consider and execute appropriate actions, communication refers to how clinical information is conveyed to other members of the team, and leadership reflects the surgeons’ maintenance of standards and control of all activities in the operating room, particularly during stressful situations [2]. Literature suggests that nearly 50% of errors contributing to medical malpractice claims result from poor communication (i.e., 53% of poor communication events were attributed to provider-patient communication breakdowns and 47% were communication issues between providers) [3]. In surgery, specifically, NTS errors have been linked to technical errors and reduced patient safety [4]. Indeed, 43% of surgical errors during patient care are attributable to communication errors among healthcare personnel [5]. In a study of 45 attending surgeons’ NTS in the clinical environment over a period of 7 months, researchers have found that increased surgeon NTS (i.e., evaluated using observer-based ratings) are associated with reduced risk of patient mortality, post-operative complications, and return to the operating room [6]. Given the importance of NTS for clinical performance, it is necessary to consider how surgeons’ NTS are currently evaluated.

Current gold standards to measure NTS in surgery involve observer-based assessments in the clinical environment [7]. Observer-based measures of NTS rely on behavioral anchors to categorize the dynamic behaviors performed by providers in the clinical environment into numeric values [8]. However, due to the complex and subtle nature of surgeon NTS in the clinical environment, extensive rater training may be needed to accurately and reliably utilize common NTS measures [9]. Furthermore, by nature, NTS are highly interrelated. For example, research indicates that the communication of pertinent patient or case information underlies all interpersonal and cognitive NTS [10]. Isolating the evaluation of specific NTS in the clinical environment may be challenging. It is necessary, then, to consider assessment methods that allow for consistent and specific evaluation of surgeons’ NTS.

In healthcare education, simulation has emerged as an effective modality to provide learners with reproducible and immersive experiences [11]. Simulation, consisting of both procedural skills training and scenario-based learning opportunities, affords learners the opportunity to practice skills on inanimate models without risking harm to patients [11–15]. Simulation has also shown promise as an effective method for high-stakes examination of trainees’ clinical skills. For example, the Advanced Cardiac Life Support program consists of a series of scenario-based simulations that evaluate incoming resident physicians’ ability to manage emergent cardiac events and is used as a certification tool for residents [16]. Despite the evidence suggesting simulated scenarios can aid in trainees’ acquisition of NTS, there have been few attempts, if any, to develop scenarios with embedded measures of NTS [17–20]. Indeed, even in simulated scenarios, NTS are evaluated with the same global NTS assessments utilized in the clinical environment [8]. Given the controlled and reproducible environment afforded through simulated scenarios, it may be possible to develop events that evaluate specific NTS constructs. However, current simulation-based approaches to evaluating NTS do not isolate specific NTS for evaluation and do not have event-specific behavioral anchors for NTS evaluation.

The purpose of the current study was to identify simulated surgical patient care scenarios and events that discretely and effectively measure NTS constructs and appropriately measure NTS using specific behavioral anchors. We aimed to identify these simulated events by establishing consensus among researchers with expertise in evaluating surgeons’ NTS.

## Methods

**Fig. 1:**
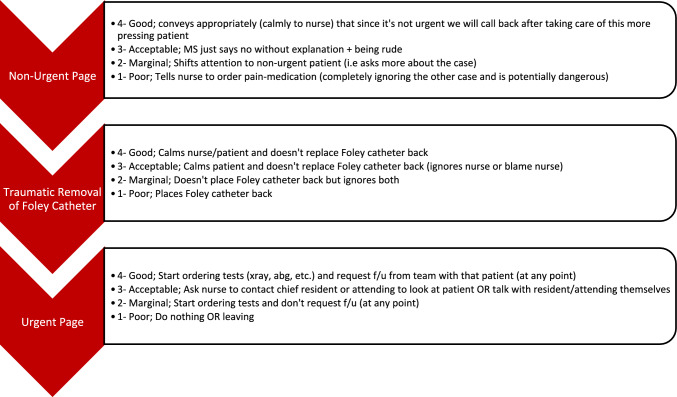
Example NTS simulation events with embedded anchors

Our team, which has expertise in simulation scenario design, has developed a large library of scenarios with specific behavioral anchors to measure NTS [19–21]. Accordingly, the research team first compiled a comprehensive list of all scenarios designed by our team to measure or train practicing surgeons, surgery residents, or medical students in NTS. These scenarios and behavioral anchors were inspired by our team’s collective expertise in evaluating surgeons’ NTS in the clinical intraoperative environment and the Non-Technical Skills for Surgeons (NOTSS) framework [22–25]. All scenarios were designed with a presenting pre-operative, intraoperative, or post-operative clinical problem that participants had to manage. All cases focused on a single participant portraying the role of primary resident or surgeon in charge of the scenario, and all other members of the team were embedded to ensure consistency and replicability. Furthermore, the patient in all cases was represented by a high-fidelity patient manikin (Sim Man 3 g, Laerdal Medical). The same patient manikin was used for all cases. Throughout these scenarios, unexpected challenges to surgeons’ NTS were introduced by embedded research team members and evaluated by trained raters after the scenario using discrete behavioral anchors for each event. The behavioral anchors were developed specifically for each event by members of the research team. The goal of these anchors was to measure NTS constructs on 3 or 4-point scales ranging from poor to exemplary for that skill. Each point of the scales included exemplar behaviors that represented that respective level of NTS performance to guide raters in their evaluation of trainees (Fig. 1).

In round one, a team of six individuals (i.e., human factors PhD Students and surgical education research fellows and researchers, with significant experience assessing NTS in the clinical and simulation environment) performed a preliminary evaluation of scenarios and events on their suitability for further evaluation based on their appropriateness to challenge an NTS domain (i.e., defined in our scenarios as either Situation Awareness, Decision Making, Communication, or Leadership) and the effectiveness of the behavioral anchors to distinguish good and poor NTS. Suitable scenarios and events would be chosen for further evaluation in round two. In round two, two additional raters with expertise in surgeon NTS (i.e., human factors psychologist and surgeon) and who were not involved in the development of the simulated events were recruited to evaluate the suitable events identified in round one. Experts completed a survey to evaluate the appropriateness and effectiveness of identified events and met virtually to discuss ratings further and identified the most effective simulated events to assess surgeons’ NTS.

### Round one

First, a framework was provided to reviewers that offered a comprehensive explanation of the NTS domains, explanations of sub-categories within each domain, and examples of exemplary and poor surgeon behaviors for each sub-category. At this time, the scenarios and NTS events were also provided to reviewers. This included a total of six scenarios with 30 discrete events. The following week, a virtual meeting was scheduled to review the framework, address any outstanding questions, and review the NTS scenarios and events in light of this framework. Each scenario stem was read to reviewers before reviewing each component event and the behavioral anchors to measure the NTS construct. Each reviewer was encouraged to provide feedback on the events. When a consensus was reached on whether an event should be included in the second round of evaluation, the team moved on to the next event.

### Round two

In round two, a survey was developed using REDCap electronic data capture tools that provided respondents with the simulation case stem (i.e., including the case location, supplies available, and initial script provided to participants) [26, 27]. The survey has been provided in Appendix A. Then, each event was provided with the specific NTS construct being challenged, the behavioral anchors and their accompanying scores for the event, and a brief video clip showing the event occurring in the simulation environment. Respondents were then asked to rate the appropriateness of the event to measure the defined NTS construct on a five-point scale: 1—“Disagree Completely”, 2—“Disagree”, 3—“Neutral”, 4—“Agree”, 5—“Agree Completely”, and the effectiveness of the behavioral anchors to measure the defined NTS construct for this event on the same scale. Respondents were also provided optional free-text response options for both measures for each event.

The two raters were provided the NTS framework developed for round 1 via electronic mail and instructed to ask the facilitator any questions before proceeding to event rating. The experts then rated each of the events using the provided scale. Following event ratings, a virtual meeting was held to discuss any discrepancies in ratings and come to a consensus on which three events were optimal to study NTS in simulation scenarios.

### Statistical analyses

The Statistical Package for Social Sciences (version 29) was used for statistical analysis (International Business Machines Corporation, Armonk, NY). Interrater agreement in round two evaluations was measured using the coefficient of variation (CV) for each event and overall agreement. The CV, or the proportion of the standard deviation to the mean, is calculated using the following formula: $\text{CV}= \frac{\sigma }{\mu }$. The CV has been identified as an appropriate measure of interrater agreement for quantitative data [28]. A CV closer to 0 represents better interrater agreement, and results can be interpreted as: 0–0.7 = excellent agreement, 0.07–0.13 = moderate agreement, and > 0.13 = low agreement. Furthermore, since our objective was to establish a consensus among raters on those simulated events with high appropriateness and effectiveness ratings and raters completed evaluations independently, this approach represents a pseudo-Delphi study design. Based on a Delphi study with more than 100 respondents per round across three rounds, the CV was identified as the best statistical procedure to establish agreement in this type of research [29]. The current study differs appreciably from this previous work (i.e., two raters, only implemented in a single round), but since our objective was to measure the consensus between raters, we elected to use the CV in our work.

## Results

The six raters in round one identified 14 events over five scenarios for further evaluation in round two (Appendix B). In round 2, the average event ratings for appropriateness of events for raters was 3.9 and 3.6, respectively. Regarding the effectiveness of NTS behavioral anchors, average event ratings were 3.8 and 3.2, respectively. Overall agreement between raters was moderate (CV = 0.12). The overall CV ranged from 0 to 0.33. The CV for appropriateness of events to measure NTS was 0.11, and the CV for effectiveness of NTS behavioral anchors was 0.14. The CV for each event is presented in Table 1. Further inspection revealed that 86% of event ratings (i.e., for both appropriateness of events and effectiveness of anchors) had discordance within ± 1 point.

**Table 1:** Coefficient of variation for appropriateness and effectiveness ratings for events

Event	Appropriateness CV	Effectiveness CV
1	0.2	0.2
2	0	0.2
3	0	0.14
4	0.33	0.33
5	0.14	0.14
6	0	0.33
7	0	0
8	0	0.14
9	0.14	0
10	0.33	0.14
11	0.11	0.11
12	0.11	0
13	0.11	0.14
14	0	0

### Appropriateness of events

**Fig. 2:**
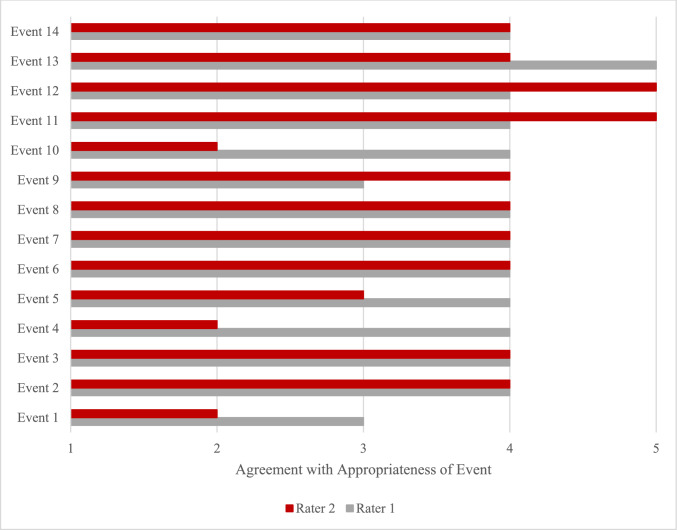
Ratings for appropriateness of events to measure NTS construct

Out of ten possible points (i.e., the combined score of the two raters), three events had the highest score of 9 out of 10 (Fig. 2). These events included event 11 (i.e., guidance of the scrub tech to secure the liver retractor for a hiatal hernia repair), event 12 (i.e., guidance of the scrub tech to maintain adequate retraction and hold the laparoscope appropriately), and event 13 (i.e., guide trainee to correct an injury that the trainee causes).

### Effectiveness of behavioral anchors

**Fig. 3:**
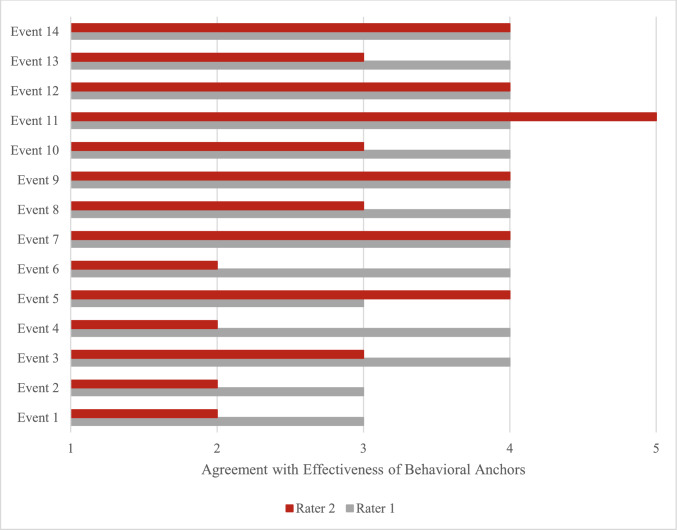
Ratings for effectiveness of behavioral anchors to assess NTS construct

Regarding the effectiveness of behavioral anchor ratings, the raters identified five events with effective ratings (i.e., events with combined scores of 8 or above) (Fig. 3).Specifically, these included events 7 (i.e., communication with anesthesia to troubleshoot causes for decreased spO2.), 9 (i.e., during timeout, novice circulator reads off incorrect procedure), 11, 12, and 14 (i.e., surgeon asks distracting personnel to leave operating room during crisis). Furthermore, the anchors for event 11 received the highest combined score of 9.

## Discussion

The purpose of this study was to systematically identify simulated scenarios that appropriately evaluate specific NTS constructs and have discrete behavioral anchors that effectively measure surgeons’ NTS. Accordingly, our team initially reviewed all simulations evaluating NTS in our surgical education research program and identified those specific events (i.e., that were designed to challenge NTS) suitable for further evaluation. Our team then invited experts in NTS from surgery and human factors to review and score the identified simulated events. Overall agreement between raters was moderate (CV = 0.12). The literature suggests that CV between 0.07 and 0.13 reflects moderate agreement between raters [28]. We also found that six of the fourteen events had absolute agreement between raters regarding their appropriateness to measure particular NTS constructs. This approach to NTS event identification is the first of its kind, as surgical education researchers may develop simulation-based curricula to study and train surgeons’ NTS without establishing expert consensus of their appropriateness and effectiveness before deployment with participants [30]. The current study consulted expert opinion to identify which NTS simulation events and behavioral anchors could serve as effective simulation-based assessments of surgeons’ NTS.

Of the 14 events that were rated, 9 of 14 events were rated highly, but 3 events stood out having highly rated appropriateness and effectiveness to measure NTS (i.e., events 11, 12, and 14). These events focused on interpersonal NTS constructs like leadership and communication. These findings align with the literature on NTS evaluation, as interpersonal behaviors are easier and more reliably captured through explicit behaviors compared to cognitive NTS like decision-making and situation awareness (i.e., evidenced by higher reliability in the Non-Technical Skills for Surgeons tool interpersonal skill ratings compared to more variable ratings of cognitive skills) [31].

Reviewing our findings in more depth, it appears that raters evaluated events that clearly isolated single NTS constructs highly, whereas events that measured multiple NTS constructs concurrently were rated poorly. For example, event 11 was rated as a 9 out of 10 (i.e., indicating complete agreement) for its appropriateness to measure NTS and the effectiveness of its behavioral anchors, and one of the raters commented “I like this example for leadership”. The event specifically involved the need to place a liver retractor for a procedure and the first assist/trainee does not have experience affixing/constructing the liver retractor and is unable to do so independently. This event is realistic, as static liver retractors are commonly placed in minimally invasive surgical procedures but are not commonplace in all specialties [32]. Furthermore, due to operating room staffing shortages in hospitals across the United States, first assists may be required to assist in specialties outside of their primary specialty [33, 34]. For this event, ideal leadership is defined as the surgeon educating the first assist on how to place the retractor, whereas suboptimal leadership is defined as the surgeon becoming frustrated and exhibiting signs of stress. The NOTSS provides a framework for exemplary surgical leadership and details it as setting and maintaining standards, coping with pressure, and supporting others [25]. This event clearly isolates leadership of the OR team through participants’ support of the staff and ability to cope with pressure.

As opposed to the clear isolation and evaluation of surgical leadership in event 11, some events were more convoluted and were rated poorly accordingly. Event 1, for instance, was the lowest-rated event regarding its appropriateness to evaluate an NTS construct. One rater commented that this event did not isolate the assessment of surgeons’ leadership and instead, “addresses several of the other NTS”. The interrelation of NTS constructs has been suggested by researchers in the past. Indeed, in the aviation industry, the non-technical skills scale (NOTECHS) to evaluate crew members’ NTS opted not to include communication in their assessment due to the belief that it was inherent to all other aspects of NTS and was difficult to isolate [10, 35]. Our developed simulations faced similar challenges regarding the isolation of NTS for evaluation, which was most impactful to poorly rated events.

Three events (i.e., 4, 6, and 10) had high CVs compared to the others, which reflects poor rater agreement on their appropriateness and effectiveness. The high variability in ratings has several possible explanations. First, event 4, which had high CV for appropriateness and effectiveness, requires participants to manage an urgent page about a deteriorating patient in a different room (i.e., while caring for the primary deteriorating patient) requires surgeons to allocate resources between the two patients and make a decision about which patient is more acute. It is possible that this particular scenario is more appropriate to measure decision-making than leadership. Furthermore, the anchors may not effectively capture good and acceptable decisions about whether to attend to the urgent page or the primary patient, which led to the discordance in ratings. Event 6, which had high CV for just the effectiveness of the anchors, was designed to measure surgeons’ situation awareness and communication regarding the deterioration of the patient’s vital signs intraoperatively. However, the anchors we used focused more on surgeon communication in response to this event rather than their situation awareness. The lack of specificity of these anchors to measure situation awareness may have contributed to the high variability in responses. Finally, event 10 had high CV for the appropriateness of the simulation to measure situation awareness and leadership. The event was designed to simulate a patient falling off the operating room table due to not being secured properly. However, since the surgeon was expected to join the case after the patient was draped and did not have the opportunity to verify the appropriateness of the patient’s security before repositioning them, these circumstances may have artificially decreased the participants’ situation awareness. This could have contributed to the discordance in appropriateness evaluations between raters.

There were limitations to the current study. Rater agreement between experts was only moderate. Compared to studies that report interrater agreement for NTS evaluations, such as the NOTSS, rater agreement is often higher (e.g., intraclass correlation coefficients among trained NOTSS raters has been reported at 0.72 for the leadership domain) [36]. However, unlike studies involving actual NTS evaluations, there was no calibration training for our raters prior to establishing consensus. We relied entirely on their perceptions about the appropriateness and effectiveness of our simulated events to capture and measure surgeons’ NTS without any interference from the research team. Similar to a Delphi survey approach, which solicits expert opinions asynchronously to avoid any potential bias from impacting expert opinions, we wanted experts to review the simulated events without any interference from the study team [37]. However, unlike a Delphi approach which attempts to conduct multiple rounds of ratings to obtain a consensus, we relied on a single virtual meeting to verify findings and reach consensus. Despite this limitation, the percentage of ratings within one point of each other was 86%, which suggests that the expert raters were generally aligned in their evaluation of NTS events.

Another potential limitation of our study was the use of just two expert raters to evaluate events in round 2. In an effort to limit bias, our team aimed to recruit NTS experts outside of our study team, as this would ensure they did not participate in developing the scenarios. Due to these logistical constraints, our team was limited in the number of raters we were able to recruit for round 2. While the limited number of raters may have reduced the overall evaluation variability that would otherwise be seen with additional raters, the two selected raters represented expertise from the fields of surgery and human factors. Thus, the raters had diverse backgrounds, which may have contributed to their moderate absolute agreement. We can be confident, then, that those events that were evaluated highly by both raters represent appropriate and effective events for the measurement of surgeons’ NTS. Another limitation of our study was the measurement of appropriateness of the scenarios to measure particular NTS constructs and the effectiveness of behavioral anchors to delineate between various levels of NTS. We utilized five-point Likert scales to measure the salient aspects of simulated events to our study objectives, but there is no established literature supporting this approach. That being said, our overall study approach did align with established literature on the design of simulation scenarios to support performance assessment in healthcare [38]. Specifically, our team developed scenarios and mapped them to specific NTS constructs for surgeons in accordance with the literature; we selected a validation team with expertise in simulation and NTS measurement to review the content and identify scenarios to include for further review, and established consensus on the appropriateness and effectiveness of simulated events among experts using independent evaluation to avoid introducing bias.

While this initial work to identify simulated scenario that can effectively and appropriately measure NTS constructs is valuable, rigorous measurement science approaches will be needed to establish the utility of these simulated events to measure surgeons’ NTS. Specifically, establishing the interrater reliability of these NTS events will be paramount to determine if the detailed behavioral anchors contribute to consistent ratings. In order to establish the interrater reliability of these events and anchors, our team plans to conduct an initial study with at least three raters to establish kappa across ratings. Unlike assessing the proportion of absolute agreement between raters, which is crude given the lack of consideration to chance agreement, kappa enables researchers to study rater agreement while accounting for chance agreement [39]. This rigorous approach to studying interrater reliability of this proposed NTS approach is needed.

## Conclusions

In this novel study, our team systematically developed simulation scenarios and specific events that enable the evaluation of surgeons’ NTS. The expert raters involved in our study identified three events that were appropriate for the evaluation of surgeons’ leadership and communication. In the future, our team may be able to leverage these scenarios to evaluate the benefits of NTS interventions or identify objective measures of surgeons’ NTS.

## Supporting data

The author confirms that all quantitative data generated or analyzed during this study are included in this published article. Supporting qualitative data will be made available upon reasonable request.

## Supplementary Information

Below is the link to the electronic supplementary material.

Supplementary file1 (PDF 69 KB)

Supplementary file2 (DOCX 19 KB)
