# Supplementary material for: Development of simulation scenarios for surgeons’ non-technical skills evaluation
Source: Global Surg Educ. 2025 Sep 13;4(1):79. doi: 10.1007/s44186-025-00390-6 (PMC12433441; doi:10.1007/s44186-025-00390-6)
Supplement: Supplementary file 1 — Supplementary file1 (PDF 69 KB) [file 44186_2025_390_MOESM1_ESM.pdf]

NTS Event Review

The purpose of this work is to attempt to achieve consensus on our assessment of surgeons' non-technical skills inside and outside the operating room (e.g., emergency department consult). Your role in this project will be to 1) evaluate current events that are being used to measure NTS in our surgical simulations on their appropriateness and sensitivity to measure specific NTS constructs and 2) consider other methods of evaluating surgeons' NTS.

For part 1, we will ask you to review simulation case stems for context, and then review video clips of our established events and rate their appropriateness to capture the specified NTS construct on a 5 point Likert scale. We will also ask you to review the behavioral anchors for NTS ratings and rate their sensitivity on a 5 point Likert scale. Finally, we would sincerely appreciate any feedback you have on any of the rating anchors or NTS events themselves, which can be provided using the optional free text boxes at the end of each event.

Case 1 Stem:

Set up: Male Mannequin on ED stretcher.

Supplies: IV pole, IV fluids and NG tubing, linens for bed.

Script: Hello, is this the Surgery Intern? I am calling about a patient, Mr. Gomez, He is a 53 yo male who had presented to the ED with 14 hours of abdominal pain and was admitted to the surgical service with a diagnosis of small bowel obstruction. Since admission, his pain has been stable, he was being treated conservatively with NPO, NG tube and IV fluids. A few minutes ago, he called me into his room because he had severe abdominal pain which woke him up from sleep. I tried giving him pain medication, but he had no relief. I am really worried about him would you mind coming to evaluate him?

Patient has a NG tube in place. Never had pain like this before. Has not passed gas or had bm since admission

Abdominal film: pneumoperitoneum (or acute abdominal series, or lateral decubitus/upright chest)

NTS constructs being assessed: Leadership

Event 1: Non-Urgent Page

Participant is paged and told, "I am calling because there is a patient who is not happy with his pain medication regiment. He says he normally gets 1mg of Dilaudid and sometimes fentanyl and his Norco isn't cutting it". Occurs as participant is starting their initial assessment of the patient.

Specific NTS challenged: Leadership

Anchors:

4- Good;conveys appropriately (calmly to nurse) that since it's not urgent we will call back after taking care of this more pressing patient

3- Acceptable;MS just says no without explanation + being rude

2- Marginal;Shifts attention to non-urgent patient (i.e asks more about the case)

1- Poor;Tells nurse to order pain-medication (completely ignoring the other case and is potentially dangerous)

1) This event is appropriate to measure the defined NTS construct.

◯ Disagree Completely
◯ Disagree
◯ Neutral
◯ Agree
◯ Agree Completely

2) Please provide any additional comments on your appropriateness rating for this event

____

3) The behavioral anchors effectively measure the defined NTS construct for this event.

◯ Disagree Completely
◯ Disagree
◯ Neutral
◯ Agree
◯ Agree Completely

4) Please provide any additional comments on your sensitivity rating of the behavioral anchors for this event

____

Event 2: X-Ray Call

When the student calls for x-ray, nurse will call them and convey that they have a few other chest xrays to take care of first but will be there in a few minutes. Participant needs to f/u on xray, they either call again or continue to wait. Participant should assign someone to f/u with xray.

Specific NTS challenged: Leadership

Anchors:

4- Good;Orders x-ray STAT and ask someone to follow-up or do so themselves if time passes by (radiologists send a page saying they will be there in 1min but they never come...)

3- Acceptable;Orders x-ray STAT but doesn't follow-up/needs to be prompted about it (nurse: hey I wonder what is going on with the x-ray) à then calls

2- Marginal;follows-up after the second prompt

1- Poor;never follow-up with the x-ray (even following 2nd prompt)

5) This event is appropriate to measure the defined NTS construct.

◯ Disagree Completely
◯ Disagree
◯ Neutral
◯ Agree
◯ Agree Completely

6) Please provide any additional comments on your appropriateness rating for this event

____

7) The behavioral anchors effectively measure the defined NTS construct for this event.

◯ Disagree Completely
◯ Disagree
◯ Neutral
◯ Agree
◯ Agree Completely

8) Please provide any additional comments on your sensitivity rating of the behavioral anchors for this event

____

Event 3: Foley Catheter is Pulled

Foley catheter gets pulled by nurse who becomes hysteric. Patient is screaming in pain. Participant is expected to calm nurse down, evaluate patient, call Urology, handle stressful event by projecting calm.

Specific NTS Challenged: Leadership

Anchors:

4- Good;Calms nurse/patient and doesn't replace Foley catheter back

3- Acceptable;Calms patient and doesn't replace Foley catheter back (ignores nurse or blame nurse)

2- Marginal;Doesn't place Foley catheter back but ignores both

1- Poor;Places Foley catheter back

9) This event is appropriate to measure the defined NTS construct.

- ◯ Disagree Completely
- ◯ Disagree
- ◯ Neutral
- ◯ Agree
- ◯ Agree Completely

10) Please provide any additional comments on your appropriateness rating for this event

____

11) The behavioral anchors effectively measure the defined NTS construct for this event.

- ◯ Disagree Completely
- ◯ Disagree
- ◯ Neutral
- ◯ Agree
- ◯ Agree Completely

12) Please provide any additional comments on your sensitivity rating of the behavioral anchors for this event

____

Event 4: Urgent Page

Page goes off/call (patient acute issue (really sick), participant is expected to assign roles and allocate resources (Attending or chief) to see that patient)

Specific NTS Challenged: Leadership

Anchors:

4- Good;Start ordering tests (xray, abg, etc.) and request f/u from team with that patient (at any point)

3- Acceptable;Ask nurse to contact chief resident or attending to look at patient OR talk with resident/attending themselves

2- Marginal;Start ordering tests and don't request f/u (at any point)

1- Poor;Do nothing OR leaving

13) This event is appropriate to measure the defined NTS construct.

- ◯ Disagree Completely
- ◯ Disagree
- ◯ Neutral
- ◯ Agree
- ◯ Agree Completely

14) Please provide any additional comments on your appropriateness rating for this event

____

15) The behavioral anchors effectively measure the defined NTS construct for this event.

- ◯ Disagree Completely
- ◯ Disagree
- ◯ Neutral
- ◯ Agree
- ◯ Agree Completely

16) Please provide any additional comments on your sensitivity rating of the behavioral anchors for this event

____

Case 2 Stem:

Set up: Mannequin on ED stretcher Chest tube in place.

Supplies: IV pole, IV fluids and chest tube pleuravac, linens for bed. A water container in the room

Script: Hello, Is this the Surgery Intern? I am calling from the Emergency Department. I have a Trauma consult for you. He is a 31-year-old male unrestrained driver involved in a Motor vehicle collision. He was initially evaluated as a trauma 3 by us here in the ED. On primary survey patient's airway was intact. His O2 saturation was 82 and was found to have decrease breath sounds on the right, chest xray showed a large hemopneumothorax so a chest tube was placed. He was otherwise hemodynamically stable with a GCS of 15. I am calling because he is going to need to be admitted now that he has a chest tube. He has gotten 2 Litters of crystalloids on route and since arriving.

NTS being evaluated: Situation Awareness, Leadership

Event 5: Nurse proposal of incorrect diagnostic test

Novice nurse proposes ordering MRI to evaluate chest tube placement based on recent paper they read, which is asinine to manage issue given criticality of situation. Participants are expected to calmly express that isn't appropriate and request different test. Ask follow up questions, explain why certain test is more appropriate than what they're suggesting.

Specific NTS challenged: Situation Awareness, Leadership

Anchors:

4- Good; Asks F/u questions to nurse to get their perspective, conveys appropriately (calmly to nurse) why test is inappropriate for current case

3- Acceptable; Acknowledging nurse suggestion but saying no without explanation

2- Marginal; Not acknowledging nurse suggestion and ordering something else

1- Poor; Going along with nurse suggestion of inappropriate test or medication

17) This event is appropriate to measure the defined NTS construct.

◯ Disagree Completely
◯ Disagree
◯ Neutral
◯ Agree
◯ Agree Completely

18) Please provide any additional comments on your appropriateness rating for this event

____

19) The behavioral anchors effectively measure the defined NTS construct for this event.

◯ Disagree Completely
◯ Disagree
◯ Neutral
◯ Agree
◯ Agree Completely

20) Please provide any additional comments on your sensitivity rating of the behavioral anchors for this event

____

Case 3 Stem:

60 yo female with PMHx of HTN, diabetes, COPD, and smoking presented to the ER 2 hours ago with c/o acute abdominal pain in the past day that she has not had before, nausea, fever, and feeling weak. Patient is hard stick and several ER nurses attempted but were unable to get an IV in her. The ER physician had therefore to place a right IJ central line that allowed them to give her fluids, which leads to pneumothorax evolving throughout case. Participant is asked to take over for surgeon who began procedure but is feeling too ill to continue.

NTS being evaluated: situation awareness, decision making, communication

Event 6: Patient Vitals Begin to Deteriorate

Vital sign monitor alarm begins to sound, alerting surgeon to decreased spO2. Surgeons are expected to attend to alarm quickly and inquire with anesthesia about reason for deterioration.

Specific NTS challenged: Situation Awareness, Communication

Anchors:

2- Ideal;listens well, closed loops, discusses the possibilites with Ax and plans next steps.

1- Acceptable;listens to the information but no closed looping or consideration of options, not sharing the decision and not leading the team. Surgeon may just start looking around the surgical field for an issue.

0- Suboptimal;Does not respond/review the possibilities with Anesthesiologist and lead the diagnostic plan.

21) This event is appropriate to measure the defined NTS construct.
- ◯ Disagree Completely
- ◯ Disagree
- ◯ Neutral
- ◯ Agree
- ◯ Agree Completely

22) Please provide any additional comments on your appropriateness rating for this event

____

23) The behavioral anchors effectively measure the defined NTS construct for this event.
- ◯ Disagree Completely
- ◯ Disagree
- ◯ Neutral
- ◯ Agree
- ◯ Agree Completely

24) Please provide any additional comments on your sensitivity rating of the behavioral anchors for this event

____

Event 7: Surgeon troubleshooting with anesthesia further

Surgeon should communicate with anesthesia to troubleshoot causes for decreased spO2.

Specific NTS challenged: Communication, Decision Making

Anchors:

2- Ideal;Surgeon follows up with Ax and closes loop on update. IF Ax does not follow up.

1- Acceptable;Surgeon requests follow up on vitals within 3 mins IF Ax does not follow up.

0- Suboptimal;Surgeon does not request follow up on vitals within 3 mins IF Ax does not follow up.

25) This event is appropriate to measure the defined NTS construct.
- ◯ Disagree Completely
- ◯ Disagree
- ◯ Neutral
- ◯ Agree
- ◯ Agree Completely

26) Please provide any additional comments on your appropriateness rating for this event

____

27) The behavioral anchors effectively measure the defined NTS construct for this event.
- ◯ Disagree Completely
- ◯ Disagree
- ◯ Neutral
- ◯ Agree
- ◯ Agree Completely

28) Please provide any additional comments on your sensitivity rating of the behavioral anchors for this event

____

Event 8: Decision that Patient is Suffering Pneumothorax.

Surgeons should exercise effective decision making to consider central line placed in ED and potential for pneumothorax. Then should communicate concern for pneumothorax with all members of the team and request chest tube.

Specific NTS challenged: Decision Making, Communication

Anchors:

2- Ideal;Surgeon recognizes possible tension pneumo, communicates that and continues to gather more information (asks anesthesiologist to reassess breath sounds, asks about central line in ED).

1- Acceptable;Surgeon recognizes and communicates concern for tension pneumo.

0- Suboptimal;Surgeon does not communicate concern for pneumothorax without being prompted.

29) This event is appropriate to measure the defined NTS construct.

◯ Disagree Completely
◯ Disagree
◯ Neutral
◯ Agree
◯ Agree Completely

30) Please provide any additional comments on your appropriateness rating for this event

____

31) The behavioral anchors effectively measure the defined NTS construct for this event.

◯ Disagree Completely
◯ Disagree
◯ Neutral
◯ Agree
◯ Agree Completely

32) Please provide any additional comments on your sensitivity rating of the behavioral anchors for this event

____

Case 4: Standard Laparoscopic Hiatal Hernia Repair

Surgeon performing laparoscopic hiatal hernia repair with Toupet fundoplication for patient with GERD

Scenario participants: surgeon (define if attending, resident level etc), circulator

Embedded participants: (anesthesiologist, trainee circulator, scrub tech)

Environmental: The patient presents in the OR, induced and prepared for laparoscopic surgery; the team is ready for you to scrub in and join them.

NTS being evaluated: Situation Awareness, Leadership, Communication

Event 9: Nurse Reads off Incorrect Procedure

During timeout, novice circulator reads off incorrect procedure (s/he reads hernia repair with Nissen fundoplication). Surgeon is expected to identify and correct to correct procedure.

Specific NTS Challenged: Situation Awareness, Leadership

Anchors:

2- SA: Surgeon recognizes and corrects/Lead: Nurse educates on procedure verification.

1- SA: Surgeon recognizes and corrects gets upset or makes ironic comment /Lead: Nurse recognizes and corrects the mistake in an abrupt manner and does not educate the novice.

0- Surgeon doesn't recognize procedure is incorrect and proceeds (if this happens, anesthesia pipes up and asks what case this is?), Nurse doesn't recognize and correct the mistake and does not educate the novice circulator on how to verify before procedure

---

33) This event is appropriate to measure the defined NTS construct.

◯ Disagree Completely
◯ Disagree
◯ Neutral
◯ Agree
◯ Agree Completely

---

34) Please provide any additional comments on your appropriateness rating for this event

____

---

35) The behavioral anchors effectively measure the defined NTS construct for this event.

◯ Disagree Completely
◯ Disagree
◯ Neutral
◯ Agree
◯ Agree Completely

---

36) Please provide any additional comments on your sensitivity rating of the behavioral anchors for this event

____

Event 10: Patient is not secured on table properly.

Prior to requesting patient position change, surgeon should confirm patient has been properly secured to OR table.

Specific NTS challenged: Situation Awareness, Leadership

Anchors:

2- Identifies patient's leg falling off table, provides clear, closed-loop communication to get patient's leg back on table and secured; maintains composure.

1- Identifies patient's leg falling off table but does not follow closed loop comm when getting patient's leg back on table and secured; gets upset

0- Not verifying that patient is appropriately secured on table prior to requesting position change and missing the right leg falling off table (50% sliding off?)

37) This event is appropriate to measure the defined NTS construct.

- ◯ Disagree Completely
- ◯ Disagree
- ◯ Neutral
- ◯ Agree
- ◯ Agree Completely

38) Please provide any additional comments on your appropriateness rating for this event

____

39) The behavioral anchors effectively measure the defined NTS construct for this event.

- ◯ Disagree Completely
- ◯ Disagree
- ◯ Neutral
- ◯ Agree
- ◯ Agree Completely

40) Please provide any additional comments on your sensitivity rating of the behavioral anchors for this event

____

Event 11: Placement of Liver Retractor.

Surgeon needs to place a liver retractor to expose the hiatus. Scrub tech tries to help secure the liver retractor to the table but is unable to do so.

Specific NTS Challenged: Leadership

Anchors:

2- Surgeon either positions retractor themselves while verbally walking through process to educate scrub, or has scrub position the retractor with guided instruction

1- Surgeon affixes retractor themselves without any education to scrub

0- Surgeon becomes frustrated (becomes short, raises voice, moves hurriedly)

41) This event is appropriate to measure the defined NTS construct.

- ◯ Disagree Completely
- ◯ Disagree
- ◯ Neutral
- ◯ Agree
- ◯ Agree Completely

42) Please provide any additional comments on your appropriateness rating for this event

____

43) The behavioral anchors effectively measure the defined NTS construct for this event.

- ◯ Disagree Completely
- ◯ Disagree
- ◯ Neutral
- ◯ Agree
- ◯ Agree Completely

44) Please provide any additional comments on your sensitivity rating of the behavioral anchors for this event

____

Event 12: Scrub needs to assist surgeon.

There is no assistant for case. Scrub needs to assist surgeon and does not maintain adequate retraction/hold scope appropriately 2x. Surgeon is expected to calmly redirect scrub and effectively communicate how they are to assist effectively.

Specific NTS challenged: Leadership, Communication

Anchors:

2- Surgeon maintains composure (politely requesting adjustment) throughout the scenario

1- Surgeon maintains composure (politely requesting adjustment) initially but becomes increasingly frustrated with repeated instances of poor assistance

0- Surgeon becomes frustrated (becomes short, raises voice, moves hurriedly)

45) This event is appropriate to measure the defined NTS construct.

- ◯ Disagree Completely
- ◯ Disagree
- ◯ Neutral
- ◯ Agree
- ◯ Agree Completely

46) Please provide any additional comments on your appropriateness rating for this event

____

47) The behavioral anchors effectively measure the defined NTS construct for this event.

- ◯ Disagree Completely
- ◯ Disagree
- ◯ Neutral
- ◯ Agree
- ◯ Agree Completely

48) Please provide any additional comments on your sensitivity rating of the behavioral anchors for this event

____

Case 5 Stem:

Surgeon performing laparoscopic cholecystectomy

Scenario participants: surgeon (define if attending, or senior resident), circulator

Embedded participants: (anesthesiologist, scrub tech, resident)

Environmental: The patient presents in the OR, induced and prepared for laparoscopic surgery; the team is ready for the surgeon to scrub in and join them.

NTS assessed: Situation Awareness, Decision Making, Communication, Leadership

Event 13: Trainee Causes Injury

Trainee causes injury and has difficulty following instructions afterwards due to high stress. Surgeon is expected to calmly take over for trainee to correct issue in a timely fashion or provide appropriate guidance to trainee to do so themselves without losing their composure.

Specific NTS challenged: Situation Awareness, Decision Making, Communication

Anchors:

2- Surgeon calmly takes over for trainee and corrects issue in a timely fashion or provides appropriate guidance to trainee to do so themselves without losing their composure

1- Surgeon takes over abruptly in a timely fashion and/ or provides a plan to address the bleeding but loses their temper and screams at trainee for causing injury

0- Surgeon does not act / provide guidance how to address the bleeding within 5 seconds

49) This event is appropriate to measure the defined NTS construct.
- ◯ Disagree Completely
- ◯ Disagree
- ◯ Neutral
- ◯ Agree
- ◯ Agree Completely

50) Please provide any additional comments on your appropriateness rating for this event

____

51) The behavioral anchors effectively measure the defined NTS construct for this event.
- ◯ Disagree Completely
- ◯ Disagree
- ◯ Neutral
- ◯ Agree
- ◯ Agree Completely

52) Please provide any additional comments on your sensitivity rating of the behavioral anchors for this event

____

Event 14: Interruption during injury by outside personnel.

Anesthesia colleague comes in during trainee injury event and begins discussing weekend plans with anesthesiologist. Surgeon is expected to calmly explain situation and request that they leave the OR.

Specific NTS challenged: Leadership

Anchors:

2- Surgeon calmly explains situation and asks anesthesiologist to remain vigilant for vital sign changes

1- Surgeon asks colleague to leave but does not provide justification to have anesthesiologist focus on vital changes

0- Surgeon does not ask for full focus or yells at colleague to leave

---

53) This event is appropriate to measure the defined NTS construct.

- ◯ Disagree Completely
- ◯ Disagree
- ◯ Neutral
- ◯ Agree
- ◯ Agree Completely

---

54) Please provide any additional comments on your appropriateness rating for this event

____

---

55) The behavioral anchors effectively measure the defined NTS construct for this event.

- ◯ Disagree Completely
- ◯ Disagree
- ◯ Neutral
- ◯ Agree
- ◯ Agree Completely

---

56) Please provide any additional comments on your sensitivity rating of the behavioral anchors for this event

____
