# Supplementary material for: Development of simulation scenarios for surgeons’ non-technical skills evaluation
Source: Global Surg Educ. 2025 Sep 13;4(1):79. doi: 10.1007/s44186-025-00390-6 (PMC12433441; doi:10.1007/s44186-025-00390-6)
Supplement: Supplementary file 2 — Supplementary file2 (DOCX 19 KB) [file 44186_2025_390_MOESM2_ESM.docx]

**Appendix B. Final Events and Behavioral Anchors Selected for Inclusion in Final Evaluation**

| **Scenario** | **Event** | **Behavioral Anchors** |
| --- | --- | --- |
| Patient in ED complaining of abdominal pain | Participant is paged and told, "I am calling because there is a patient who is not happy with his pain medication regiment. He says he normally gets 1mg of Dilaudid and sometimes fentanyl and his Norco isn't cutting it". Occurs as participant is starting their initial assessment of the patient. | 4- Good; conveys appropriately (calmly to nurse) that since it's not urgent we will call back after taking care of this more pressing patient  3- Acceptable; MS just says no without explanation + being rude  2- Marginal; Shifts attention to non-urgent patient (i.e asks more about the case)  1- Poor; Tells nurse to order pain-medication (completely ignoring the other case and is potentially dangerous) |
|  | When the student calls for x-ray, nurse will call them and convey that they have a few other chest xrays to take care of first but will be there in a few minutes. Participant needs to f/u on xray, they either call again or continue to wait. Participant should assign someone to f/u with xray. | 4- Good; Orders x-ray STAT and ask someone to follow-up or do so themselves if time passes by (radiologists send a page saying they will be there in 1min but they never come...)  3- Acceptable; Orders x-ray STAT but doesn't follow-up/needs to be prompted about it (nurse: hey I wonder what is going on with the x-ray) à then calls  2- Marginal; follows-up after the second prompt  1- Poor; never follow-up with the x-ray (even following 2nd prompt) |
|  | Foley catheter gets pulled by nurse who becomes hysteric. Patient is screaming in pain. Participant is expected to calm nurse down, evaluate patient, call Urology, handle stressful event by projecting calm. | 4- Good; Calms nurse/patient and doesn't replace Foley catheter back  3- Acceptable; Calms patient and doesn't replace Foley catheter back (ignores nurse or blame nurse)  2- Marginal; Doesn't place Foley catheter back but ignores both  1- Poor; Places Foley catheter back |
|  | Page goes off/call (patient acute issue (really sick), participant is expected to assign roles and allocate resources (Attending or chief) to see that patient) | 4- Good; Start ordering tests (xray, abg, etc.) and request f/u from team with that patient (at any point)  3- Acceptable; Ask nurse to contact chief resident or attending to look at patient OR talk with resident/attending themselves  2- Marginal; Start ordering tests and don't request f/u (at any point)  1- Poor; Do nothing OR leaving |
| Patient in ED experiences decreasing O2 saturation | Novice nurse proposes ordering MRI to evaluate chest tube placement based on recent paper they read, which is asinine to manage issue given criticality of situation. Participants are expected to calmly express that isn't appropriate and request different test. Ask follow up questions, explain why certain test is more appropriate than what they're suggesting. | 4- Good; Asks F/u questions to nurse to get their perspective, conveys appropriately (calmly to nurse) why test is inappropriate for current case  3- Acceptable; Acknowledging nurse suggestion but saying no without explanation  2- Marginal; Not acknowledging nurse suggestion and ordering something else  1- Poor; Going along with nurse suggestion of inappropriate test or medication |
| Patient experiences intraoperative pneumothorax | Vital sign monitor alarm begins to sound, alerting surgeon to decreased spO2. Surgeons are expected to attend to alarm quickly and inquire with anesthesia about reason for deterioration. | 2- Ideal; listens well, closed loops, discusses the possibilites with Ax and plans next steps.  1- Acceptable; listens to the information but no closed looping or consideration of options, not sharing the decision and not leading the team. Surgeon may just start looking around the surgical field for an issue.  0- Suboptimal; Does not respond/review the possibilities with Anesthesiologist and lead the diagnostic plan. |
|  | Surgeon should communicate with anesthesia to troubleshoot causes for decreased spO2. | 2- Ideal; Surgeon follows up with Ax and closes loop on update. IF Ax does not follow up.  1- Acceptable; Surgeon requests follow up on vitals within 3 mins IF Ax does not follow up.  0- Suboptimal; Surgeon does not request follow up on vitals within 3 mins IF Ax does not follow up. |
|  | Surgeons should exercise effective decision making to consider central line placed in ED and potential for pneumothorax. Then should communicate concern for pneumothorax with all members of the team and request chest tube. | 2- Ideal; Surgeon recognizes possible tension pneumo, communicates that and continues to gather more information (asks anesthesiologist to reassess breath sounds, asks about central line in ED).  1- Acceptable; Surgeon recognizes and communicates concern for tension pneumo.  0- Suboptimal; Surgeon does not communicate concern for pneumothorax without being prompted. |
| Standard Laparoscopic Hiatal Hernia Repair | During timeout, novice circulator reads off incorrect procedure (s/he reads hernia repair with Nissen fundoplication). Surgeon is expected to identify and correct to correct procedure. | 2- Ideal; Surgeon recognizes and corrects  1- Acceptable; Surgeon recognizes and corrects gets upset or makes ironic comment  0- Suboptimal; Surgeon doesn't recognize procedure is incorrect and proceeds (if this happens, anesthesia pipes up and asks what case this is?) |
|  | Prior to requesting patient position change, surgeon should confirm patient has been properly secured to OR table. | 2- Ideal; Identifies patient's leg falling off table, provides clear, closed-loop communication to get patient's leg back on table and secured; maintains composure.  1- Acceptable; Identifies patient's leg falling off table but does not follow closed loop comm when getting patient's leg back on table and secured; gets upset  0- Suboptimal; Not verifying that patient is appropriately secured on table prior to requesting position change and missing the right leg falling off table (50% sliding off?) |
|  | Surgeon needs to place a liver retractor to expose the hiatus. Scrub tech tries to help secure the liver retractor to the table but is unable to do so. | 2- Ideal; Surgeon either positions retractor themselves while verbally walking through process to educate scrub, or has scrub position the retractor with guided instruction  1- Acceptable; Surgeon affixes retractor themselves without any education to scrub  0- Suboptimal; Surgeon becomes frustrated (becomes short, raises voice, moves hurriedly) |
|  | There is no assistant for case. Scrub needs to assist surgeon and does not maintain adequate retraction/hold scope appropriately 2x. Surgeon is expected to calmly redirect scrub and effectively communicate how they are to assist effectively. | 2- Ideal; Surgeon maintains composure (politely requesting adjustment) throughout the scenario  1- Acceptable; Surgeon maintains composure (politely requesting adjustment) initially but becomes increasingly frustrated with repeated instances of poor assistance  0- Suboptimal; Surgeon becomes frustrated (becomes short, raises voice, moves hurriedly) |
| Standard Laparoscopic Cholecystectomy | Trainee causes injury and has difficulty following instructions afterwards due to high stress. Surgeon is expected to calmly take over for trainee to correct issue in a timely fashion or provide appropriate guidance to trainee to do so themselves without losing their composure. | 2- Ideal; Surgeon calmly takes over for trainee and corrects issue in a timely fashion or provides appropriate guidance to trainee to do so themselves without losing their composure  1- Acceptable; Surgeon takes over abruptly in a timely fashion and/ or provides a plan to address the bleeding but loses their temper and screams at trainee for causing injury  0- Suboptimal; Surgeon does not act / provide guidance how to address the bleeding within 5 seconds |
|  | Anesthesia colleague comes in during trainee injury event and begins discussing weekend plans with anesthesiologist. Surgeon is expected to calmly explain situation and request that they leave the OR. | 2- Ideal; Surgeon calmly explains situation and asks anesthesiologist to remain vigilant for vital sign changes  1- Acceptable; Surgeon asks colleague to leave but does not provide justification to have anesthesiologist focus on vital changes  0- Suboptimal; Surgeon does not ask for full focus or yells at colleague to leave |
